# Using phage display technology to obtain Crybodies active against non-target insects

**DOI:** 10.1038/s41598-017-09384-x

**Published:** 2017-11-02

**Authors:** Tania Domínguez-Flores, María Dolores Romero-Bosquet, Diana Marcela Gantiva-Díaz, María José Luque-Navas, Colin Berry, Antonio Osuna, Susana Vílchez

**Affiliations:** 1 Institute of Biotechnology, Campus Fuentenueva s/n, University of Granada, 18071 Granada, Spain; 2 Cardiff School of Biosciences, Cardiff University, Park Place, Cardiff, CF10 3AT United Kingdom; 3 Department of Biochemistry and Molecular Biology I, Campus Fuentenueva s/n, University of Granada, 18071 Granada, Spain

## Abstract

The insecticidal Cry toxins produced by *Bacillus thuringiensis* (Bt) are increasingly important in the biological control of insect pests and vectors of human disease. Markets for Bt products and transgenic plants expressing their toxins are driven by their specificity, safety and the move away from chemical control agents. However, the high specificity of Cry toxins can also prove to be a limitation when there is no known Cry toxin active against a particular target. Novel activities can be discovered by screening natural Bt isolates or through modifications of the Cry proteins. Here we demonstrate the use of λ-phage displaying Cry1Aa13 toxin variants modified in domain II loop 2 (Crybodies) to select retargeted toxins. Through biopanning using gut tissue from larvae of the non-target insect *Aedes aegypti*, we isolated a number of phage for further testing. Two of the overexpressed Cry toxin variants showed significant activity against *A*. *aegypti* larvae while another induced mortality at the pupal stage. We present the first report of the use of phage display to identify novel activities toward insects from distant taxonomic Orders and establish this technology based on the use of Crybodies as a powerful tool for developing tailor-made insecticides against new target insects.

## Introduction

*Bacillus thuringiensis* (*Bt*), is characterized by its ability to produce proteins with insecticidal activity. Toxins produced by *Bt* can form crystalline inclusions during sporulation^1^ known as δ endotoxins (toxins Cry and Cyt)^2,3^ or can secrete toxins during vegetative growth (VIP and Sip toxins)^4,5^. Among these proteins, Cry toxins are the best characterized and exert their effect on the host by causing lysis of the midgut epithelial cells, which leads to gut paralysis, cessation of insect feeding, loss of membrane integrity, release of the cellular contents and eventual death^3,6–8^.

Currently, 302 holotypes of Cry toxins are described (http://www.btnomenclature.info/) as a result of a huge international effort to isolate and characterize novel *Bt* strains. Each of these toxins is reported to be active against a limited number of targets (mostly insects but also nematodes, molluscs and, in a few cases, human cancer cells^9^) and this specificity is one of the most remarkable characteristics of Cry toxins. This specificity is a consequence of their complex and sophisticated mode of action, which, although not completely understood, is well-accepted to require the presence of receptors on the epithelial membrane of the target gut cells^2,10–12^.

Although Cry toxins may belong to a number of distinct structural families^9,13^, the major group within Cry proteins is the so-called 3-domain toxins. The three-dimensional structure of these toxins shows a remarkable similarity^14^. Domain II, formed by three antiparallel beta-sheets arranged in a beta-prism form^15^, has been associated with toxin specificity^12,16^. Domain II is one of the most variable of the three domains, especially in three apical loops in this part of the molecule. These loops differ not only in sequence and conformation, but also in length. This variability, together with domain II’s similarity to the complementarity-determining region of immunoglobulins^15^ led researchers to associate this domain with the role of receptor binding and hence with specificity. Exchange of the domain II regions among Cry toxins or modification of the sequences of the loops seems to be enough to alter the toxicity profile toward an insect^17–19^.

As mentioned above, the mechanism of action of 3-domain Cry toxins is not fully elucidated and currently three different models have been postulated for their function^14,20^. All proposed mechanisms involve binding to receptors present on the surface of target cells such as cadherin-like proteins, the amino peptidase N (APN), alkaline phosphatase (ALP), glycolipids^21^, the glycoconjugate BTR-270^22^, the P252 protein^23^ and the recently described transporter ABCC2^24,25^. Although binding to the receptor is not the only requirement for toxicity, it is an essential requirement and it seems that natural evolution of Cry toxins has adapted them to recognize different proteins as receptors to be used in their mechanism of action.

The toxic effect of Cry proteins has been commercially exploited for more than 70 years to control insect populations that have an impact on forestry, agriculture and health^2,26,27^ and toxin genes have also been used in plant transgenesis^28–34^. The specificity of the *Bt* Cry toxins is a huge advantage; each toxin only affects a restricted number of insects species, leaving other, non-target organisms unaffected^35^. Unfortunately, in many cases, there is no known toxin active against the insect of interest that can be used for its control. The traditional approach to search for new activities against a specific insect has been the isolation of hundreds of new *Bt* strains, and bioassaying their activity against the target insect to probe toxicity^36–38^. This represents an extensive work programme and frequently no activity at all is found against the insect of interest.

An alternative strategy to obtain Cry toxins with novel specificities is the modification of existing toxins through molecular techniques in order to increase the activity toward certain insects^39–41^ and rationally redesign activity from one insect to another^17,19,41,42^.

Although these studies demonstrate that manipulation of toxins can render novel activities, this process is labour intensive and not very effective as many of the mutants rationally designed in this way may lack structural stability and function. Here, we demonstrate a high throughput screening methodology for the potential isolation of new toxins with novel activities against insect targets. The methodology is based on phage display technology, a potent molecular tool for *in vitro* selection of proteins with a specific binding profile. In this technique, a protein or a mutant library is fused to a phage protein so that it is displayed on the phage surface, ready to interact freely with other proteins. Several groups have described phage display of Bt toxins^43–49^ but successful isolation of variants active toward taxonomically distant non-target insects has never been reported.

Previously, we reported the display of a Cry1Ac toxin on the surface of a lambda phage that successfully interacted with its natural receptor^50^. This was followed by a report of the construction of several Cry1Aa mutant libraries by replacing loops 1, 2 and 3 from the Domain II of the toxin with the hypervariable region contained at the complementary determinant region 3 (CDR-H3) of a human antibody library^51^. The resulting cc2 library was displayed on the surface of a phage. The system combined the structure and scaffolding of the Cry toxins with the potential binding capability and specificity of a library of human antibodies (Crybodies). Here we present the screening of this library for the selection of toxins with novel binding profiles towards *Aedes aegypti*, an important vector of several flavivirus disease agents such as Dengue virus, Yellow fever virus and the currently emerging Zika virus. The selected Cry1Aa variant toxins were cloned in a heterologous expression system, overexpressed and bioassayed against *A*. *aegypti*, the non-canonical target insect chosen as a model. Cry toxin variants selected *in vitro* using the phage display showed significant toxicity to *A*. *aegypti*. Thus, we demonstrate the proof of principle that *in vitro* evolution of a Cry toxin coupled with *in vitro* selection using phage display is a powerful tool for selecting novel toxins redirected against new-target insects across insect orders.

## Results

### *In vitro* selection of phage with affinity to *A*. *aegypti* guts

The λcc2 phage display library, containing a diversity of 2.2 × 10^7^ unique clones of Cry toxins with the domain II loop 2 of the lepidopteran active Cry1Aa13 toxin replaced by CDR-H3 sequences obtained from a human antibody library^52^ was used in this study. A series of biopanning experiments was performed in order to select and enrich those phage from the library with enhanced affinity to targets present in the *A*. *aegypti* guts compared to the wild-type toxin. The pool of the phage obtained was considered as the sub-library 1. These phage were amplified and used for a new round of selection using fresh *A*. *aegypti* guts and this procedure was used in two further steps, each using the enriched phage from the previous round. The sub-library 4, resulting from the fourth round of selection, was used for further analysis. The number of phage recovered in each round of selection is shown in supplementary Table S1. As no significant increase of phage number in each round of selection was observed, further analysis was carried out in order to gain insight into any specific selection that may have occurred. To do this, the length of the loop 2 sequences present in a group of 15 randomly selected phage from sub-library 4 obtained by PCR with primers A2f and A2r, Supplementary Table 2S) was compared to the loop length from a group of 15 randomly selected clones from the original library λcc2 (Supplementary Fig. S1). The loop 2 size of phage in sub-library 4 seemed to be less variable than the original library, probably indicating that a selective process had successfully occurred.

### Sequence analysis of loop 2 variants from sub-library 4

**Figure 1:**
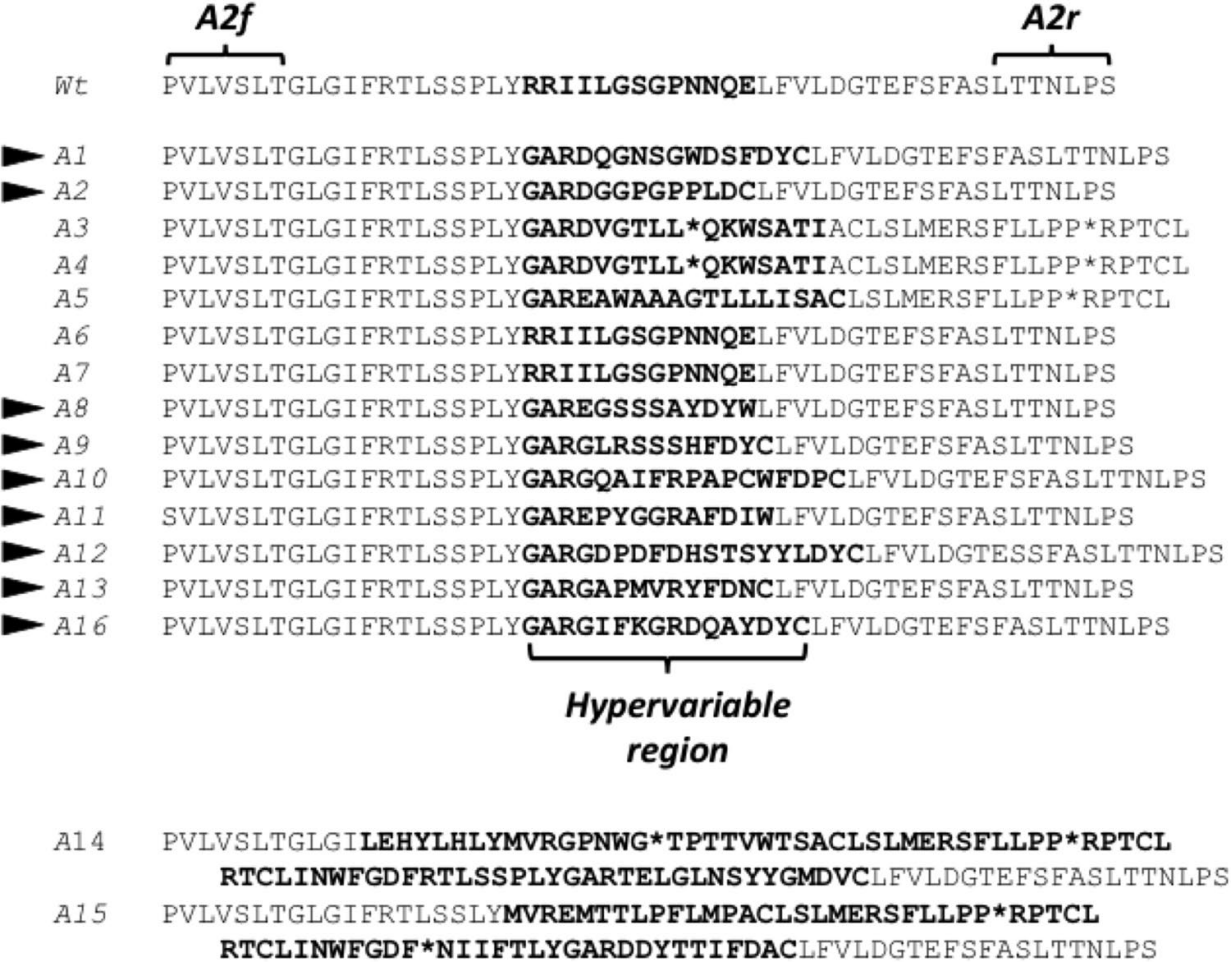
Alignment of the loop 2 sequences present in the *in vitro* selected Cry phage and the wild type Cry1Aa13 toxin. Arrowheads indicate novel loop variants in sequences that do not contain premature stop codons. Bold letters represent the predicted hypervariable region present in each mutant after loop 2 replacement.

A2f-A2r PCR fragments obtained from a further 16 phage from sub-library 4 (different from the pool analysed in Fig. S1 and named from A1 to A16) were cloned in the pGEM-T vector and the resulting plasmids were sequenced and the amino acid sequence present at the loop 2 of the selected Cry mutants was determined (Fig. 1). The sequence analysis revealed that five of the phage (A3, A4, A5, A14 and A15) might display a truncated version of Cry toxins as a stop codon was found in their sequences (shown as “*”). The existence of these truncated proteins was probably due to errors in the assembly of the library or DNA amplification that rendered a shift in the reading frame as previously reported^51^. Phage A3 and A4 showed the same sequence and the stop codon was found at the hypervariable region. Phage A14 and A15 show complex insertion patterns in which Gly-Ala-Arg- sequences introduced during library production, occur in the middle of the inserts rather than at the end. Phage A6 showed the same sequence as A7, corresponding to the wild type loop of Cry1Aa13. The rest of the phage A1, A2, A8, A9, A10, A11, A12, A13 and A16 (Fig. 1, black arrows) showed a sequence completely different to each other and different from the wild type with no stop codons introduced.

The number of aa present at the hypervariable region in these 9 selected phage ranged from 13 to 19 aa. Compared to the original λcc2 library (7 to 25 aa reported by^51^), the variability in sizes decreased significantly, probably due to the selection process.

The 9 loop-variant mutants that did not contain stop codons (A1, A2, A8, A9, A10, A11, A12, A13 and A16), began with either Asp, Arg or Gly (after the Gly-Ala-Arg- sequence introduced during library production). Seven of the 9 ended with Cys (the other two ending with Trp), Tyr was the penultimate residue in 5 of these variants, preceded by Asp in 8 of the variants, preceded by an aromatic Phe (5 variants) or Tyr (2 variants). Whether this reflects a positive selection for these residues during biopanning or a feature of the original library is not known at present.

### Reconstituting Crybodies from the selected phage

Phage in the library contain translational fusions between the active part of Cry1Aa13 toxin (containing loop variants) and the phage coat protein gpD to allow toxin to be displayed on the phage surface^50,51^. In order to assay the toxin variants selected with *A*. *aegypti* guts, loop variants were recloned into the pCP10-Δloop2 plasmid (following the strategy described in the Materials and Methods section and summarized in Supplementary Fig. S2). The resulting constructs were PCR screened with A2f and TD2 primers (Supplementary Table S2) in order to select those clones with the correct orientation of the fragment. Plasmids pCP10-A1, pCP10-A2, pCP10-A5, pCP10-A8, pCP10-A9, pCP10-A10, pCP10-A11, pCP10-A12, pCP10-A13 and pCP10-A16 (named collectively as pCP10-A*i*) were produced and contained mutant *cry1Aa13* protoxin genes from phage A1, A2, A5, A8, A9, A10, A11, A12, A13 and A16 respectively.

### Expression profile of the cloned Crybodies

**Figure 2:**
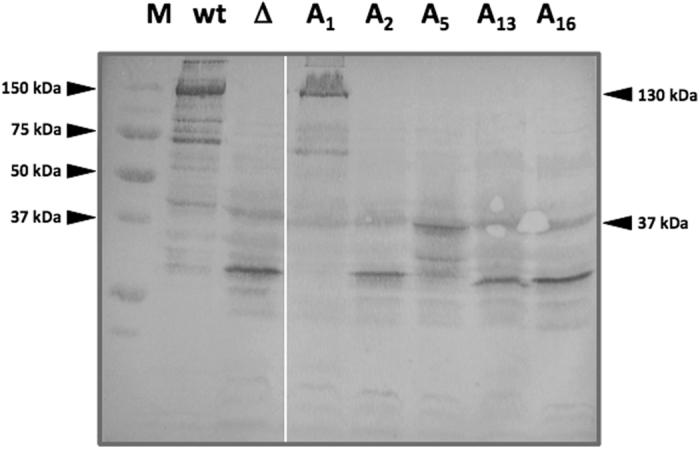
Production of variant protoxins. Coomassie blue stained 12% acrylamide gel showing the insoluble fractions of IPTG induced cultures of *E*. *coli* with pCP10 (lane wt) as positive control, and pCP10-Δ loop2 (lane Δ) as negative control. Clones bearing plasmids pCP10-A1 (lane A1), pCP10-A2 (lane A2), pCP10-A5 (lane A5), pCP10-A13 (lane A13), pCP10-A16 (lane A16), and pCP10-A9 (lane A9), did not show over-expression of the Cry protoxin. Clones bearing plasmids pCP10-A8 (lane A8), pCP10-A10 (lane A10), pCP10-A11 (lane A11) and pCP10-A12 (lane A12) showed a strong Cry protoxin production similar to the wild type Cry protoxin.

*E*. *coli* clones containing pCP10-A*i* plasmids were grown in liquid media with IPTG and the accumulation of the mutant protoxins was analysed by SDS-PAGE (Fig. 2). In the pellet fraction of an *E*. *coli* pCP10 culture, a protein of 130 kDa corresponding the wild type Cry1Aa13 protoxin was observed (Fig. 2, lane 1). No such protein was detected in the clone bearing pCP10-Δloop2, as it contains a deleted version of the *cry1Aa13* gene (Fig. 2, lane 2) and hence a truncated Cry protein is produced (that is probably unstable and does not accumulate). Clones pCP10-A8, -A10, -A11 and -A12 showed a strong band of 130 kDa in size corresponding to Crybodies Cry1Aa13-A8, -A10, -A11, and -A12 respectively. Clone pCP10-A1 (Fig. 2, lane 3) produced a minor band at this size. The rest of the clones did not render any visible protoxin.

Those clones with no visible accumulation of the full Cry protoxin were analysed by western blot (Supplementary Fig. S3) and expression of the full protoxin was only observed in the clone pCP10-A1, although at a very low level, consistent with the results from Coomassie staining. In the rest of the clones, smaller proteins were detected by immunoanalysis, indicating that protoxin was expressed but the protein was not stable enough to render the full Cry toxin, probably because of the loop replacement.

### Solubilisation and trypsin activation of Cry1Aa13-A8, -A10, -A11, and -A12 Crybodies

**Figure 3:**
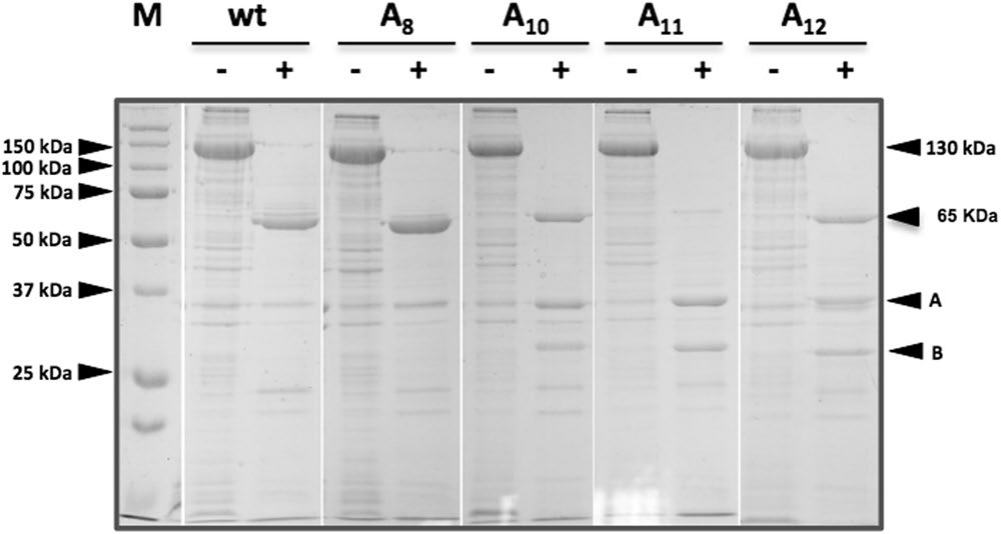
Solubilization and trypsin treatment of protoxin variants. Coomassie blue stained 12% acrylamide gel showing solubilized protein treated (+) and untreated (−) with trypsin. Cry1Aa13 wild type was used as positive control for solubilisation (lane wt −) and trypsin activation (lane wt +). Lanes A8, A10, A11 and A12 show the solubilized Cry1Aa13-A8, Cry1Aa13-A10, Cry1Aa13-A11 and Cry1Aa13-A12 respectively. Trypsin-treated toxins exhibited a protein of 65 kDa corresponding to the core of the toxin or activated toxin. Cry1Aa13-A11 showed the same band but less intense than the rest of the mutants. Arrows A and B indicate the molecular weight of the approximately 37 kDa and 28 kDa fragments observed during trypsin digestion.

The four Crybodies showing a good level of expression and stability (Cry1Aa13-A8, -A10, -A11 and -A12) were solubilized and activated with trypsin under standard *in vitro* conditions^51^. As shown in Fig. 3, all four Cry1Aa13-A*i* Crybodies were successfully solubilized under the same conditions as the Cry1Aa13 wild type (Fig. 3, lane 1) and rendered a 130 kDa protein. Solubilized Cry1Aa13-A8, -A10, and -A12 Crybodies treated with trypsin produced a band around 65 kDa corresponding to the theoretical molecular weight of the active Cry toxin^52^. However, Cry1Aa13-A11 showed a faint band around 65 kDa and more intense protein fragments of 37 and 28 kDa than the other mutants and the wild type toxin (probably as a result of digestion of the 65 kDa protein), indicating a lower stability of this mutant compared to the rest. As the stability of Cry1Aa13-A11 was compromised, this variant was not used in further experiments.

### Evaluating the toxicity of selected Crybodies against *A*. *aegypti*

**Figure 4:**
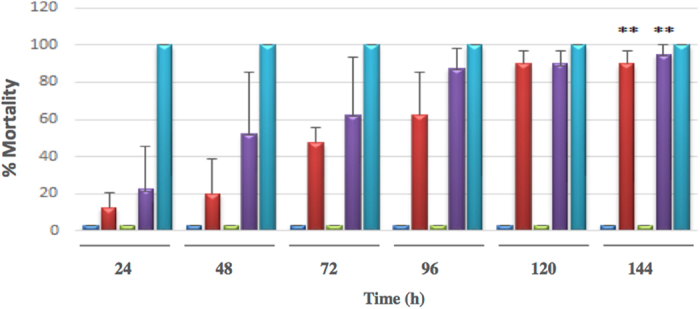
Percentage mortality of *A*. *aegypti* larvae on treatment with soluble toxins. Results obtained with Cry1Aa13-wt toxin (dark blue), Cry1Aa13-A8 (red), Cry1Aa13-A10 (green) and Cry1Aa13-A12 (purple) and *Bti* (cyan) treatments.

The susceptibility of *A*. *aegypti* larvae to mutant toxins Cry1Aa13-A8, -A10 and -A12 was determined. Figure 4 shows the larval mortality obtained in the bioassays with each toxin. The mixture of Cry4Aa, Cry4Ba, Cry11Aa and Cyt1Aa obtained from *Bti* showed 100% mortality 24 h after the beginning of the bioassay as expected. The lepidopteran-active Cry1Aa13 toxin did not produce any mortality as *A*. *aegypti* is not susceptible to this protein. However, two mutant toxins, Cry1Aa13-A8 and -A12, showed a significant percentage of mortality after 120 h (around 90% mortality).

The statistical analysis in a pair-wise comparison revealed significant differences between the toxicity of the proteins assayed. Each toxin was included in one of the 3 significantly different groups; Cry1Aa13-wt and Cry1Aa13-A10 were included in group A, Cry1Aa13-A8 and -A12 in group B, and *Bti* was included in group C. The Kaplan-Meier estimator (α = 0.05) was determined for Cry1Aa13-A8 and -A12 rendering values of 82 ± 0.87 h and 64.4 ± 0.88 h respectively. These results demonstrate that the two *in vitro* selected mutants Cry1Aa13-A8 and -A12 showed toxicity against *A*. *aegypti*, presumably mediated through affinity towards proteins present in the insect gut. LC_50_ values of these two variants were calculated by performing bioassays against 4^th^ instar larvae, using toxin concentrations ranging from 20 μg/ml to 1.25 μg/ml. Using Probit analysis it was estimated that LC_50_ values for Cry1Aa13-A8 and Cry1Aa13-A12 were 10.4 μg/ml and 9.1 μg/ml respectively (Table 1). For comparative purposes, Table 1 also details the LC_50_ of the naturally mosquitocidal toxins Cry4Aa, Cry4Ba, Cry11Aa and CytAa toxins reported in the bibliography^19,53–58^ and reviewed by Otieno-Ayayo *et al*.^59^. Taking the highest LC_50_ values for each natural toxin we can observe that Cry1Aa13-A8 showed a LC_50_ approximately 4.2 times higher than Cry4Aa, 10 times higher than Cry4Ba and Cyt1Aa and 35 times higher than Cry11Aa. In the case of Cry1Aa13-A12, the LC_50_ was 3.75, 10, 31 and 9 times higher than Cry4Aa, Cry4Ba, Cry11Aa and Cyt1Aa respectively.

**Table 1:** LC_50_ values of Cry1Aa13 variants obtained in *A*. *aegypti* bioassays and comparison with the reported in the literature for individual toxins from *Bti*.

Toxin	LC_50_^a^ (μg/ml)	SD	Source^b^
Cry1Aa13-A8	10.4 (8.358–12.453)	1.0	This study
Cry1Aa13-A12	9.1 (8.094–10.021)	0.5	This study
Cry1Aa13-A10	≫20^a^	ND	This study
Cry1Aa13	≫20^a^	ND	This study
**Toxin**	**LC**_**50**_^**c**^ **(μg/ml)**	**Source** ^**b**^	
Cry4Aa	0.56	Poncet *et al*.^53^	
	2.39	Boonserm *et al*.^54^	
Cry4Ba	0.06	Abdullah *et al*.^19^	
	0.94	Angsuthanasombat *et al*.^55^	
Cry11Aa	0.011	Revina *et al*.^56^	
	0.28	Poncet *et al*.^53^	
Cyt1Aa	0.88	Thiery *et al*.^57^	
	1.00	Juarez-Perez *et al*.^58^	

^a^Mean lethal concentration (LC_50_) was estimated by Probit analysis. 95% confidence limits are given in parentheses when known. ND = not determined. ^b^Source of the LC_50_ values presented in the table, determined in this study or taken from literature and reviewed by Otieno-Ayayo *et al*.^59^. ^c^LC_50_ values (the lowest and the highest) reported in the literature.

### Evaluating the toxicity of selected Crybodies against *Bombyx mori*

In order to determine how loop 2 replacement influenced the activity of the selected Crybodies against a canonical insect target of the wild type, parental toxin, variants Cry1Aa13-A8, Cry1Aa13-A10 and Cry1Aa13-A12 were bioassayed against *B*. *mori* together with the wild type toxin Cry1Aa13 and a mixture of non-active toxins (Cry4, Cry11 and Cyt) from *Bti*. The results showed (Table 2) that after 96 h the Cry1Aa13-A10 variant (which is not active against *A*. *aegypti* larvae (Fig. 4)) showed 100% mortality in *B*. *mori* bioassays, as did the Cry1Aa13 wild-type toxin. In contrast, mortality produced by Cry1Aa13-A8 and Cry1Aa13-A12 variants was lower (45 ± 7% and 50 ± 14% respectively) than the wild-type, suggesting that loop 2 replacement negatively influenced in the activity against its natural lepidopteran target. As expected, low toxicity was recorded (20%) when *Bti* toxins were assayed against *B*. *mori*.

**Table 2:** *B*. *mori* mortality recorded after 96 h in contaminated mulberry leaf-bioassays.

	Toxin (36 ng/cm^2^)				
	Cry4 and Cry 11 from *Bti*	Cry1Aa13 (wt)	Cry1Aa13-A8	Cry1Aa13-A10	Cry1Aa13-A12
% Mortality ± SD	20 ± 10	100 ± 0	45 ± 7	100 ± 0	50 ± 14

### Unexpected activities observed in the selected Crybodies

In addition to these results, another interesting observation was made in the bioassay performed with the Cry1Aa13-A10 mutant. Although the mortality of the larvae was 0% at the end of the bioassay (Fig. 4) as described above, and all larvae became pupae, it was observed that all of these insects died within 24 h after pupation. This was an unexpected activity that is worth noting as, to our knowledge, pupal mortality as a result of exposure to *Bt* 3-domain Cry toxins has not been reported previously. This result also demonstrates that completely new activities can be obtained by using the phage display methodology that we have employed. Further investigations of this novel activity will be published separately.

## Discussion

It has been demonstrated extensively that the specificity of the 3-domain Cry toxins can be mediated by domain II of the protein, since this region has a role in toxin binding to natural receptors^60–64^. Mutations within the loops of domain II can induce an increase of activity against target insects^39–41^, and deletions, substitutions or replacements can even redirect toxicity toward related species^19,65^. In addition, in a program of rational redesign, the lepidopteran-active Cry1Aa was rendered toxic to the dipteran *Culex pipiens* by specific replacements in loops 1 and 2 of domain II^42^. As these studies involved rational redesign, similar experiments are only feasible when assessing the effects of chimeric proteins made from toxins with known activities and not for the development of toxicity against species that are not targets of currently available toxins. These reports demonstrate that an important determinant of 3-domain Cry toxin specificity resides in the domain II loops and open the possibility of creating new toxin activities by manipulation of these loops. Construction of high-diversity Cry toxin mutant libraries through combinatorial techniques is relatively straightforward, the challenge in this artificial evolution is selecting and identifying those mutants from the library that may show interesting activity against a specific target. Individually bioassaying each artificially-generated mutant from a combinatorial library to determine activity is impossible from a practical point of view and phage display offers a potential method for bio-panning such libraries to enrich for specific binding activities against any selected insect target. Of course, not all the variants selected would be likely to show activity, but as binding to insect guts is a requirement for Cry toxicity, binding was considered to be an appropriate filter to allow selection of a subset of variants for detailed analysis.

The display of Cry toxins on the surface of phage has been attempted using several systems, which have sometimes encountered limitations. In the first attempt to display a Cry toxin on the surface of a phage (Cry1Aa on M13 phage), the toxin was not properly displayed, resulting in deletions of the fusion protein^45^. Later, a complete Cry1Ac was displayed on the surface of the M13 phage, although the toxin did not show binding to its natural APN receptor *in vitro*, suggesting structural constraints of the displayed toxin^46^. Display systems based on λ and T7 phage proved to be more suitable alternatives for displaying Cry toxins, as they assemble in the *E*. *coli* cytoplasm and are released by cell lysis instead of secreting their components through the bacterial membrane as in M13^66^. Cry1Ac was fused to the capsid protein gpD of λ phage and successfully displayed on the phage surface^50^ with the Cry1Ac1::gpD fusion showing similar toxicity to the wild type and interacting with its natural receptors. Pacheco *et al*.^44^ reported the fusion of Cry1Ac toxin to the capsid protein 10B of T7 phage, successfully displaying a *M*. *sexta* active toxin able to bind *M*. *sexta* BBMV^44^. This T7 display system was the basis for the construction of a mutant library containing 5 × 10^5^ variants bearing a 7-8 aa-long random sequence at loop 2 of the Cry1Aa1 toxin^47^. Screening of this mutant library against *Bombyx mori*, a natural target of the parental Cry1Aa protein, identified a variant four times more potent than the wild type Cry1Aa1 but screening of the library against other insects was not reported. Recently, Craveiro *et al*. 2010 reported the selection of 4 variants of the lepidopteran-active Cry1Ia12 obtained by DNA shuffling and phage display technology. The Cry1Ia12 variants showed between 2 and 3 times more mortality than the wild type Cry1Ia12 toxin against the non-target lepidopteran sugarcane giant borer *Telchin licus licus*. This report showed that phage display can be used for selecting novel activities against new, taxonomically-related targets, but the selection of variants active against more distinct orders of insects was not reported. Thus, nearly two decades after the first publication of Cry toxin phage display, the production through this technology of toxins retargeted toward taxonomically distant targets has not been described.

The library used in our work, exploited our previous λ gpD-fusion phage display system^50^ to express loop 2 domain II variants of Cry1Aa13^51^. Compared to the display system developed by Ishikawa *et al*.^47^, this λcc2 library contained a higher number of mutants (2.2 × 10^7^ *vs* 5 × 10^5^), the variants contained loops with a wider range of mutant sequences (7–25 aa *vs* 7–8 aa) and our library was constructed by replacing loop 2 by an antibody CDR-H3 library, which may prove an inherently better source of binding motifs than artificially-generated random sequences. All these factors may underlie the ability of the system developed in the present work to provide the breakthrough to yield variants with completely novel activities.

After 4 cycles of biopanning with *A*. *aegypti* guts, the derived pool of phage showed a hypervariable region in loop 2 ranging from 13 to 19 aa as opposed to the 7–25 aa present in the original library^51^, a reduction in diversity consistent with a selection/enrichment process. The analysis of the sequences of the selected phage showed that some displayed a truncated version of the mutant toxin, probably due to assembly problems in the construction of the λcc2 library. The truncated Cry toxins are not expressed beyond domain II loop 2 and it is not clear whether their presence in the enriched phage pool is due to unexpected specific binding or whether they are part of the inevitable background binding. The presence of such phage in the library and their retention represents a decrease in the recovery efficiency of the system that we are currently working to circumvent.

Analysis of full-length Cry protoxins, reconstituted from selected phage showed that not all the Cry mutants retained during enrichment were suitable for heterologous over-expression. Attempts to express mutant toxins such as Cry1Aa13-A1, -A2, -A5, -A9, -A13, and -A16 resulted in no significant accumulation of the protoxin. As all toxins were expressed from the same promoter (P_*tac*_ promoter), the failure to accumulate these toxins is most likely due to the low stability of the mutants. This was confirmed in western blots, which detected small fragments of Cry toxins, implying that these mutant proteins were expressed but were subject to degradation. The lack of stability can only be attributed to the change in the sequence of the loop 2 as the rest of the molecule was unchanged. Low stability and increased proteolytic sensitivity observed for some mutants may arise from conformational changes elsewhere in the protein, induced in the molecule by the novel loop 2 variants (Supplementary Fig. S4). Alternatively, the introduction of random sequences may provide direct proteolytic target sites within loop 2. Mutants Cry1Aa13-A9, -A13 and -A16, which are particularly unstable and the more stable Cry1Aa13-A11 mutant may illustrate proteolytic sensitivity at the mutated loop as they contain Lys and or Arg residues in this region that would introduce potential susceptibility to cleavage by trypsin-like enzymes. Cry1Aa13-A11 is stable enough to be produced in the pellet fraction of recombinant *E*. *coli* but can be subsequently degraded by trypsin digestion, possibly at the Arg residue in its loop 2. In contrast, while the stable mutant Cry1Aa13-A10 also has an Arg residue in the loop, it appears to be activated normally by trypsin. However, the Arg in this variant is followed by a Pro residue and endoproteolytic cleavage N-terminal to a Pro residue is very unusual in nature^67^. Three mutants, Cry1Aa13-A8, -A10, and -A12 showed high levels of expression and normal trypsin-activation profiles, so they were selected for bioassays against *A*. *aegypti*. Cry1Aa13-A10 showed an unexpected activity, with no apparent effect on *A*. *aegypti* larvae but exhibiting delayed mortality at the pupal stage. Blocking the development of adults through pupal mortality may represent an important new means to disrupt disease transmission. The activity of this mutant needs to be characterized further but this variant demonstrates the capacity of the strategy developed here to expand selection of toxins beyond the larval stage of the insect, and widen the possibility of targeting other stages; a feature that could represent an advantage in the control of many natural insect populations.

Variants Cry1Aa13-A8 and -A12 were able to produce a high mortality in larval populations (90%). Although the amount of toxin used in the bioassays was quite high (20 µg/ml) the wild type Cry1Aa13 showed no toxicity at this concentration, (LC_50_ for the Cry1Aa13 is higher than 100 µg/ml according our experience). The mutant activity is clearly lower than the toxicity of *Bti* crystals used as the positive control, but these contain at least three different Cry toxins (Cry4Aa, Cry4Ba, and Cry11Aa) and one Cyt protein (Cyt1Aa) with possible production of Cyt2Ba and Cry1Ca and Cry10Aa^68,69^ active against *A*. *aegypti* with different toxicity levels^70,71^. The activity of each individual toxin produced by *Bti* against *A*. *aegypti* has been determined by several research groups and compiled by Otieno-Ayayo *et al*.^59^. These authors found a high variability of the LC_50_ reported in the literature for Cry4Aa (0.56–2.39 μg/ml), Cry4Ba (0.06–0.94 μg/ml), Cry11Aa (0.01–0.28 μg/ml) and Cyt1Aa (0.8–1.0 μg/ml) toxins against *A*. *aegypti* as a consequence of factors such as where the toxins are produced, how the toxins are purified and processed or the conditions of the bioassays. Although comparison of the LC_50_ obtained in different bioassays is not completely accurate, it provides an opportunity to demonstrate that the activity of the Cry1Aa13 variants obtained by phage display may be within an order of magnitude of the toxicity of toxins naturally active against the model insect. If we compare the highest reported LC_50_ value for each individual *Bti* toxin with the Cry1Aa13-A12 variant for example, we can roughly estimate that toxicity of the latter is 3.79–32 times lower than individual mosquitocidal toxins. *Bti* toxins have evolved to target nematoceran Diptera for millions of years, allowing evolutionary optimisation of the toxins for their target. Thus, we have demonstrated significant activity for Cry1Aa13-A8 and Cry1Aa13-A12 against *A*. *aegypti* (within one order of magnitude of the natural mosquitocidal toxins). This is a good level of toxicity for proof of principle for our system, considering that variations were introduced without rational design, in only a single loop and the derived toxins have not undergone further optimisation. The level of toxicity shown by variants Cry1Aa13-A8 and Cry1Aa13-A12 was similar to the activity gained in *C*. *pipiens* in the rational design previously reported by Liu and Dean, (2006). The mutant 1AaMosq, containing a Loop 1 substitution (^311^RG^312^ by YQDL) and a Loop 2 deletion (^365^LYRRIIL^371^) plus a substitution (^376^NNQ^378^ was replaced by a G) showed a LC_50_ value for 2^nd^ instar larvae of 45.73 μg/ml while LC_50_ for *A*. *aegypti* 4^th^ instar larvae for Cry1Aa13-A8 and Cry1Aa13-A12 was 10.4 and 9.1 μg/ml respectively. In the report of Liu and Dean^42^, loop 2 in the 1AaMosq was shortened to mimic the mosquito-active Cry4B loop 2. In contrast, our selected variants Cry1Aa13-A8 and Cry1Aa13-A12 showed a loop length similar to the Cry1Aa13 parental toxin (13 and 18 aa respectively) but very different in sequence. In addition, the loop2 sequences present in mosquito-active Cry1Aa13 variants have no significant similarity to loop 2 regions from other 3-Domain Cry toxins, including the mosquitocidal toxins Cry4Aa, Cry4Ba and Cry11.

A correlation is observed in our results between the development of toxicity of Cry1Aa13-A8 and Cry1Aa13-A12 toward *A*. *aegypti* concomitant with decreases in toxicity toward *B*. *mori*. It is interesting to note that Cry1Aa13-A10, which did not show any activity against *A*. *aegypti* larvae, retained its activity against *B*. *mori*. It will be interesting to investigate the molecular targets of these new variant toxins to determine whether they are associating with known receptor types (eg cadherins, amino peptidases, alkaline phosphatases) or new classes of binding ligands. Binding studies of the three variants with *A*. *aegypti* and *B*. *mori* BBMVs are in progress.

It should be noted that the two larvicidal and one pupicidal variants described were identified from a detailed analysis initiated from only 16 randomly selected phage from our biopanning, further refined to just 9 candidates after initial sequence analysis. This represents an excellent level of efficiency and clearly incentivises the screening of further variants. In the longer term, efficiency may be enhanced by improving library quality (with fewer truncated variants) and may be aided by the introduction of an increased number of biopanning rounds prior to clone testing. Increasing the biopanning stringency between rounds would be desirable but there are problems with changing stringency when there is little or no control over the number or concentration of target molecules^72^ (as in our screening with no defined target in tissue samples and the ability of phage to select the targets to which they might bind). Further development of higher toxicity variants may also be aided by the separate screening of libraries with diversity in variable loops ∝8, 1 and 3 with the subsequent combinatorial mixing (by simple combination or, for example, through DNA shuffling techniques) of mutants showing activity.

Very recently, a report on Cry toxin evolution using PACE (phage-assisted continuous evolution) has been described^73^. In this paper authors designed a complex and outstanding *in vitro* evolution system that enables novel Cry variants to be obtained that are active against resistant insects. This work, like ours, supports the idea that *in vitro* evolution is the strongest tool for obtaining tailor-made insecticides that the market demands. However, the evolved toxins obtained by PACE showed activity against closely-related insects species and maintained a similar insecticidal spectrum as the parental *Bt* toxin. As a result, there may be only relatively minor changes in receptor molecules to which toxins must be adapted. In contrast, we have demonstrated that Crybody phage display can go even further to obtain novel variant with activities against distinct orders of insects. The work presented here has provided the proof of principle that phage display, our library and the selection strategy developed are suitable molecular approaches for selecting toxins with activity redirected to new target insects even across taxonomic orders. This technology can now be exploited to tackle new pests for which no *Bt* toxins are currently available and also as a strategy to overcome resistance that has arisen in the field.

## Materials and Methods

### Bacterial and phage strains

*Escherichia coli* strain DH5α was used for cloning and expression studies. *E*. *coli* strain Y1088 was used for phage amplification. *E*. *coli* strain BLR containing the pCP10 plasmid was used for obtaining Cry1Aa13 toxin^52^. *B*. *thuringiensis* var *israelensis* 4Q5 was used to obtain Cry4Aa, Cry4Ba, Cry11Aa and Cyt1Aa toxins. All phage used were derived from the λEMBL3 vector (Promega)^74^. The λEMBL3-pTI11 contained the plasmid pTI11 as test insert plasmid supplied in the Promega kit and used for controlling packaging efficiency. λCP2 phage displayed the wild type Cry1Aa13 toxin and the λcc2 mutant library contained approximately 10^7^ unique variants of Cry toxins (kindly provided by Prof. D. J. Ellar from the University of Cambridge)^52^.

### Phage amplification and concentration

*E*. *coli* Y1088 was grown overnight at 37 °C (240 rpm) in 5 ml of NZCYM medium (5 g/l NaCl, 2 g/l MgSO_4_·7H_2_O, 5 g/l yeast extract, 10 g/l casein enzymatic hydrolysate, 1 g/l casein acid hydrolysate) supplemented with ampicillin (100 μg/ml). After determining the A_600 nm_ of the culture, an appropriate volume, containing 10^10^ cells (assuming that 1 U of A_600 nm_ corresponds to 10^9^ cells/ml) was centrifuged and the pellet resuspended in 300 μl of SM (100 mM NaCl, 10 mM MgSO_4_, 50 mM Tris-HCl, 0.01% gelatin, pH 7.5), and mixed with a phage suspension containing 10^7^–10^8^ pfu. After 20 min incubation at 37 °C the mixture was added to 50 ml of fresh NZCYM and incubated (37 °C, 150 rpm) in a rotary shaker until lysis of the culture was completed (A_600 nm_ 0.1–0.2). Then, 1 ml of chloroform was added, incubated for a further 20 min and treated with pancreatic DNase (Sigma) and RNase (1 μg/ml, Sigma) at RT for 30 min. Solid NaCl was added to a final concentration of 1 M and incubated on ice for 1 h before being centrifuged (12000 × g) for 20 min. The supernatant was mixed with PEG 8000 (10% (w/v) final concentration), incubated for 1 h on ice and centrifuged at 12000 × g for 20 min and the pellet resuspended in 800 μl of SM. An equal volume of chloroform was added and the mixture vortexed for 30 seconds. The upper aqueous phase containing the phage suspension was recovered and stored at 4 °C until use.

### Phage quantification and phage propagation on plates

*E*. *coli* Y1088 was cultured in 50 ml of LB (plus maltose 0.2% (w/v), 10 mM MgSO_4_ and ampicillin 100 μg/ml) at 37 °C and 240 rpm until a A_600 nm_ of 0.5–0.6. Culture was pelleted, resuspended in 25 ml of 10 mM MgSO_4_ and kept at 4 °C and used within a month when needed. For phage titration, 200 μl of this *E*. *coli* Y1088 suspension was mixed with 200 μl of a phage suspension, incubated for 20 min at 37 °C, mixed with 3–4 ml of molten top agarose (50 °C) (per liter: 10 g tryptone, 5 g yeast extract, 10 g NaCl, 6 g agarose) and poured onto a LB agar plate. Plates were incubated at 37 °C overnight for plaque counting. Plaques were propagated and amplified in solid media with the use of a sterile toothpick and a LB agar plate containing top agarose with *E*. *coli* Y1088.

### *Aedes aegypti* rearing and management

*A*. *aegypti* eggs were incubated in a plastic container with dechlorinated water and ground artificial diet (commercial dry cat food) at 25 °C ± 2 °C, 70% humidity and a photoperiod (12 h light/12 h darkness). Eggs hatched within two days. Fourth instar larvae were used for biopanning experiments and bioassays.

### *In vitro* biopanning

Biopanning was performed with ten guts extracted from Virkon®-treated 4^th^ instar *A*. *aegypti* larvae. Guts were obtained by pulling from the neck and the anal segment of the larva with 2 ultra fine point tweezers. Guts were placed in cold PBS for peritrophic membrane removal and then placed in 200 µl of SM buffer supplemented with protease inhibitors (complete mini protease inhibitor cocktail, Roche®) in an Eppendorf tube. Insect tissue was disrupted with a micropestle (Sigma) and mixed with the appropriate volume of the phage suspension of λEMBL3-pTI11, λCP2 or λcc2 to give a final concentration of 10^9^ PFU/ml. Phage and insect gut homogenates were incubated for 30 min at RT and then centrifuged at 16000 × g for 10 min. Pellets were resuspended in 500 μl of washing buffer (1.5 M NaCl, 0.1% Tween and protease inhibitor) and centrifuged again under the same conditions. Washing was repeated 5 more times and the final pellet was resuspended in 200 μl of SM buffer. Phage attached to material present in the pellet fractions were recovered by incubation (20 min at 37 °C) with 200 μl of *E*. *coli* Y1088 prepared as indicated above. The mixture was placed in 50 ml of NZCYM medium for phage amplification. When the number of the selected phage (sub-library) reached more than 10^10^ PFU/ml, a new round of biopanning was started to enrich phage able to bind to the gut (and deplete the relative proportion of phage present due to non-specific binding). Four rounds of selection were performed in total and phage present in the final sub-library were kept for further experiments and analysis.

### Loop 2 cloning and sequence determination

Loop 2 mutants present in selected phage were PCR amplified using A2f-A2r primers (Supplementary Table S2), purified and ligated into pGEM®-T vector (Promega) following the manufacturer’s instructions. Plasmids were sequenced using primer M13f.

### Constructing pCP10-Δloop2 plasmid

To facilitate recovery and overexpression of protoxin forms of *cry* gene variants identified through phage screening, we constructed the plasmid pCP10-Δloop2 containing a 500 bp deletion in the *cry1Aa13* gene that included the region encoding loop 2 of the protein. This provided a toxin scaffold ready for the insertion of any of the loop 2 mutants from the selected phage and avoided the relatively inefficient amplification and cloning of ~ 2 kb encoding the entire toxin region. *Sal*I restriction sites were introduced to facilitate the cloning strategy as the introduction of this restriction site did not change the aa composition of the protein. The pCP10-Δloop2 plasmid (Fig. S1) was constructed by PCR (95 °C/5 min, 30 cycles at 95 °C/45 sec, 60 °C/45 sec, 72 °C/7 min) using pCP10 plasmid as template and the divergent primers TD12 and TD13 (Table S2). The PCR amplicon was purified (Qiagen), digested with *Sal*I (NEB), heated for enzyme inactivation and self-ligated with T4 DNA ligase (Promega). The circularized plasmid produced, pCP10-Δloop2, was then ready to accept any mutant loop 2 in the newly introduced *Sal*I site.

### Construction of new variant toxin plasmids

The region that includes the loop 2 of domain II of the different selected phage was amplified using the primers TD10 and TD11 (Table S2) by PCR (1 cycle at 95 °C/5 min, 30 cycles at 95 °C/30 sec, 63 °C/30 sec, 72 °C/30 sec and a final extension at 72 °C/5 min). PCR fragments were purified, *Sal*I digested and ligated to the *Sal*I-dephosphorylated pCP10-Δloop2 for transformation of *E*. *coli* DH5α. Successful constructs were screened by colony PCR using A2f and TD2 primers (Table S2).

### Protein expression, solubilisation and trypsin activation conditions

Each toxin variant was cultured in 50 ml of LB (plus 1 mM IPTG and 100 μg/ml ampicillin) at 37 °C, 240 rpm for 16 h. The culture was pelleted and resuspended in 1 ml of lysis buffer (50 mM Tris HCl pH 8, 500 mM NaCl, 10 mM EDTA, 5 mM β-mercaptoethanol, 0.35 mg/ml lysozyme and 5% v/v triton) and incubated for 30 min at RT. Then, 10 μg/ml DNase (Sigma), 10 μg/ml RNase (Sigma) and 6 mM MgCl_2_ were added and incubated for a further 20 min. The cell suspension was sonicated on ice (Sonifier® SLP) for 6 min with cycles (10 s on/10 s off) at full power. The homogenate was centrifuged at 12000 × g for 20 min and the supernatant discarded. The pellets were resuspended in 500 µl of 50 mM Na_2_CO_3_ pH 9.6 and 10 mM DTT, incubated for 1 h at 37 °C, centrifuged and supernatant saved. For activation, solubilized protein was digested with TPCK-treated trypsin from bovine pancreas (Sigma) at a ratio of 1:1 (w/w) at 37 °C for 1 h. Protein samples were analysed by SDS-PAGE (12%, Coomassie blue stained). Total protein content was determined by the Bradford method. Single protein content was estimated using a ChemiDoc equipment (Bio-Rad) on SDS-PAGE gels using BSA as standards.

### *A*. *aegypti* bioassays

Ten early fourth-instar *A*. *aegypti* larvae were placed in 10 ml dechlorinated water containing 20 µg/ml of each Cry1Aa13 selected mutant, the lepidopteran active Cry1Aa13-wt or a mixture of solubilised Cry4Aa, Cry4Ba, Cry11Aa and Cyt1Aa, obtained from a sporulated culture of *B*. *thuringiensis* subsp. *israelensis* strain 4Q5 (grown in T3 medium for 72 h, centrifuged at 4000 g for 20 min, and solubilized in Na_2_CO_3_ 50 mM pH 9.6 and DTT 10 mM for 1 h at 37 °C). Apart from solubilized toxins, ground dry cat food was added in each assay. Assays were performed at 26 °C. Mortality was analyzed every 24 h until all larvae died or pupated. Each toxin was assayed in duplicate and the bioassays were performed twice. The results were analyzed statistically using the XLSTAT program.

For LC_50_ determination, bioassays were performed under the same conditions using 5 concentrations (20, 10, 5, 2.5 and 1.25 μg/ml) of Cry1Aa13-A8 and Cry1Aa13-A12. Each toxin was bioassayed in duplicate and each assay repeated twice. Mortality was recorded after 6 days and LC_50_ values were calculated by Probit analysis^75^.

### *B*. *mori* bioassays

Toxicity of Cry variants against *B*. *mori* was determined using diet contamination bioassays in neonate larvae. A mulberry leaf cut in a square shape (size 3 × 3 cm) was placed in a plastic Petri dish. On the leaf, 100 μl of a toxin solution containing 3.2 μg/ml of toxin was spread with the use of a sterile L-shaped spreader, rendering a toxin concentration of 36 ng of toxin per cm^2^ of diet, (3 times higher than the LC_50_ for Cry1Aa reported in the bibliography against *B*. *mori* ^25^. Apart from the toxin variants selected by phage display, Cry1Aa13 and a mixture of Cry toxins obtained from *Bti* were used as positive and negative controls respectively. Ten neonates of *B*. *mori* were placed in each Petri dish with the help of a fine brush. Plates were kept at 25 °C and 50% humidity in an insect room and mortality was recorded daily for 96 h.

### Modelling method

The structures of Cry1Aa13 variants were modelled on the structure of Cry1Aa^76^ submitted under accession number 1CIY in the pdb using the Swiss model program^77^. The template was believed to be valid due to the fact that it contains only 2 amino acid changes compared to the Cry1Aa13 protein.

## Electronic supplementary material

Supplementary Information
