# Supplementary material for: Using phage display technology to obtain Crybodies active against non-target insects
Source: Sci Rep. 2017 Nov 2;7:14922. doi: 10.1038/s41598-017-09384-x (PMC5668233; doi:10.1038/s41598-017-09384-x)
Supplement: Supplementary file 1 — Supplementary Information [file 41598_2017_9384_MOESM1_ESM.pdf]

Suplementary data.

Title: “Using phage display technology to obtain Crybodies active against non-target insects”

Authors: Tania Domínguez-Flores[a,b], María Dolores Romero-Bosquet[a], Diana Marcela Gantiva-Díaz[a], María José Luque-Navas[a], Colin Berry[b], Antonio Osuna[a], Susana Vílchez[a,c]*.

Table S1: Number of phage recovered in each round of selection after biopanning using *A. aegypti* guts.

	Round 1	Round 2	Round 3	Round 4
**λEMBL**	$3.9x10^{6}$	$2.6x10^{4}$	$1.6x10^{3}$	$2.1x10^{4}$
**λCP2**	$6.5x10^{4}$	$2.0x10^{5}$	50	$1.4x10^{4}$
**λcc2**	$1.3x10^{5}$	$2.3x10^{4}$	$2.0x10^{2}$	$1.2x10^{4}$
**SM**	0	0	0	0

Table S2: Primers used in this work.

Primer name	Sequence	Restriction site (in bold)
**M13f**	5’ GTTTTCCCAGTCACGAC 3’	
**A2f**	5’ CCCGTACTTGTCTCATTAACTGG 3’	
**A2r**	5’ GGAAGGCAAGTTGGTCGTTAGG 3’	
**TD2**	5’ AATT**CCCGGG**CTATTCTAAATCATATTC 3’	*Sma*I
**TD10**	5’ ATGATA**GTCGAC**GGTATCCAATTCGAACAGTTTCCC 3’	*Sal*I
**TD11**	5’ TAGTGA**GTCGAC**TGTACCCCTTTGTCTATATATAGTGG 3’	*Sal*I
**TD12**	5’ GATACC**GTCGAC**TATCATAATTTGAGAATAGAGC 3’	*Sal*I
**TD13**	5’ GGTACA**GTCGAC**TCACTAGATGTAATACCGCCACAGG 3’	*Sal*I

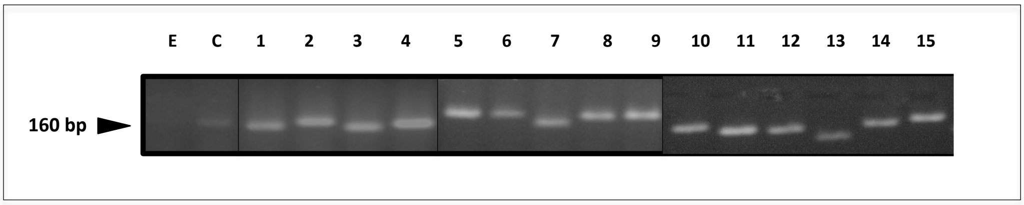

A

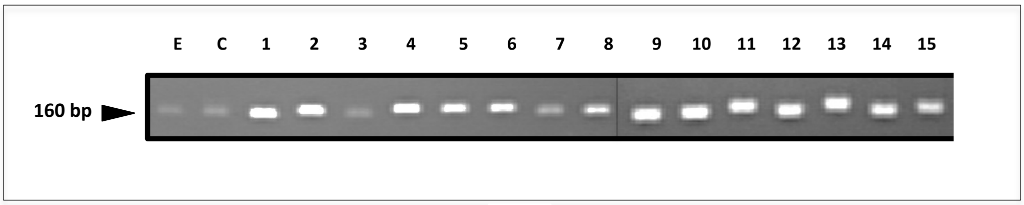

B

Figure S1: Phage library insert size variation.

Electrophoresis in 2% agarose gels of PCR fragments obtained with A2f and A2r primers showing the size of the loop 2 present in the original λcc2 library (Panel A) and in the *in vitro* selected phage (Panel B). Lane E shows the negative control (λEMBL3-pTI11) and lane C the positive control (λCP2).

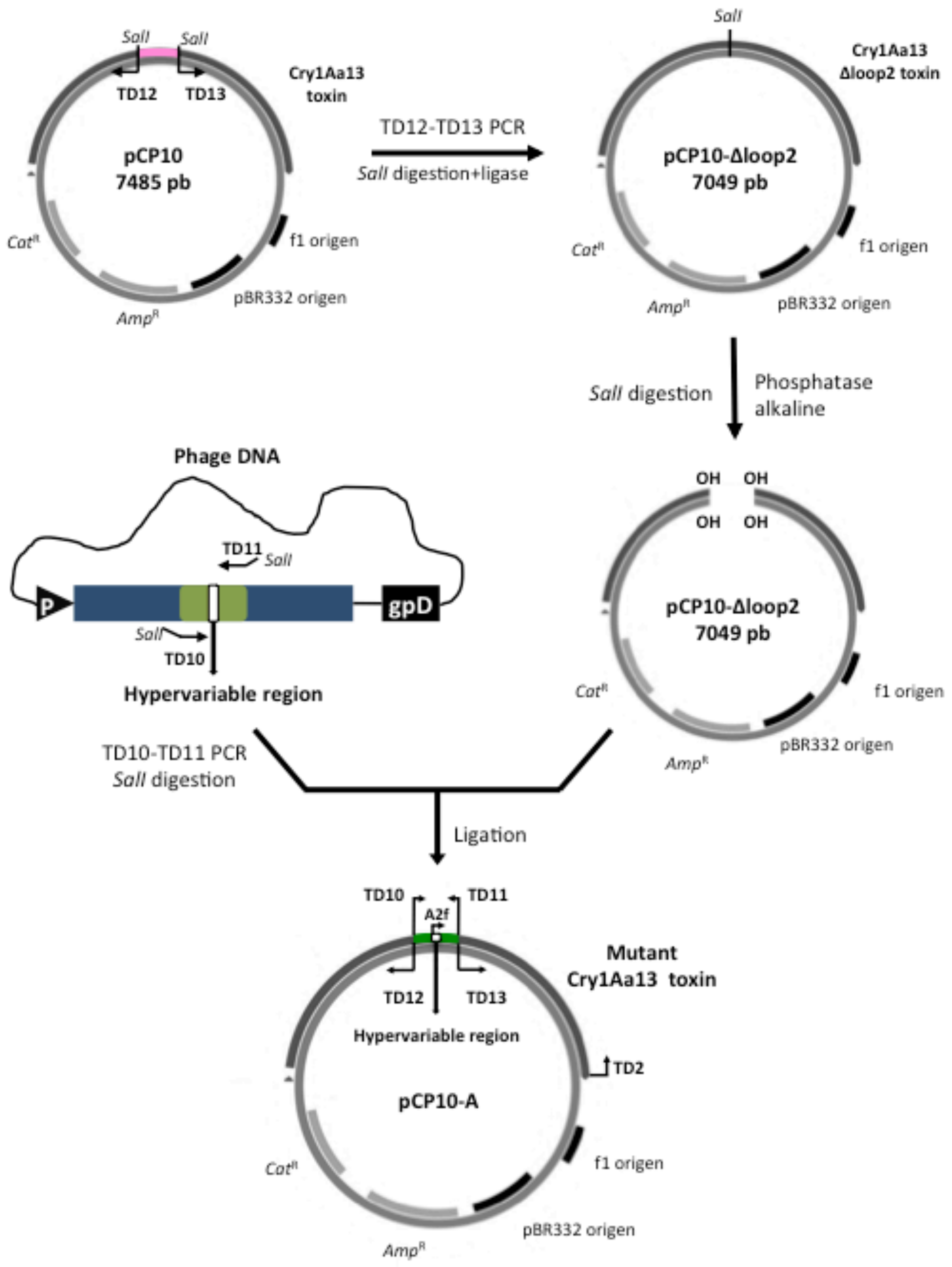

Figure S2: pCP10-Δloop2 construction and cloning strategy for the recovery of the mutant Cry toxins displayed in the selected phage.

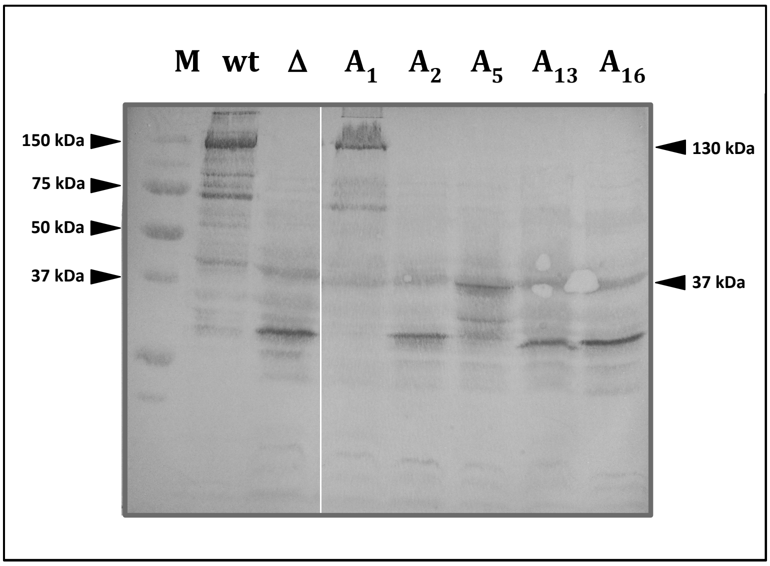

Figure S3: Immunoblot analysis of protoxin expression.

Toxin bands were revealed using a polyclonal anti-Cry1Ab antibody. Plasmid pCP10-A1 (lane A1) showed the expression of a 130 kDa protein with the same size as the positive control pCP10 (lane wt) that expresses the Cry1Aa13 protoxin. Plasmids pCP10-A2 (lane A2), pCP10-A5 (lane A5), pCP10-A13 (lane A13) and pCP10-A16 (lane A16) showed a similar protein profile as the negative control pCP10-Δloop2 (lane Δ).

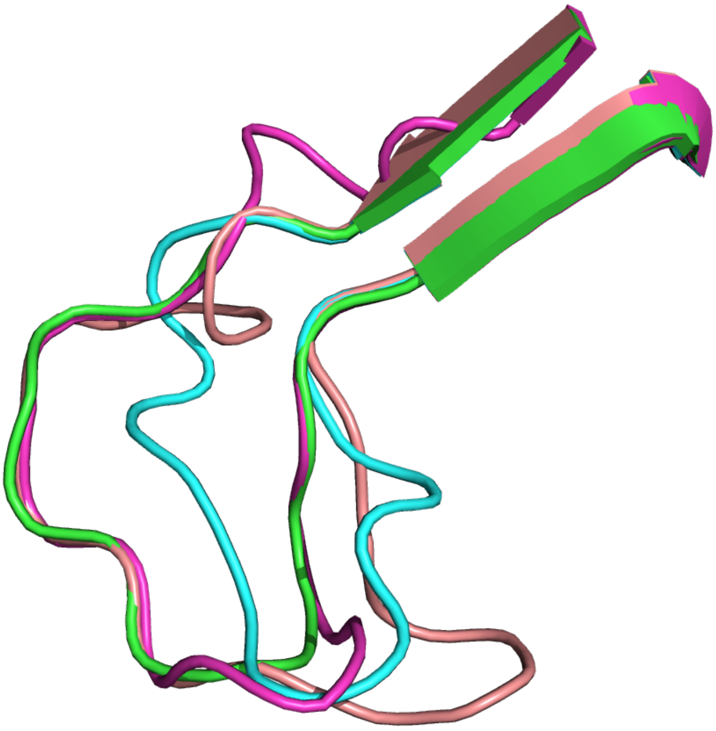

Figure S4. Modelled structure of mutant loops. The modelled structures of domain II, loop 2 for mutants Cry1Aa13-A8 (cyan), Cry1Aa13-A10 (magenta) and Cry1Aa13-A12 (pink) are overlaid on the modeled wild-type Cry1Aa13 (green) loop.
